# Supplementary material for: Liver Tumor Markers, HALP Score, and NLR: Simple, Cost-Effective, Easily Accessible Indexes for Predicting Prognosis in ICC Patients after Surgery
Source: J Pers Med. 2022 Dec 9;12(12):2041. doi: 10.3390/jpm12122041 (PMC9784982; doi:10.3390/jpm12122041)
Supplement: Supplementary file 1 [file jpm-12-02041-s001.zip › Figure S2.pdf]

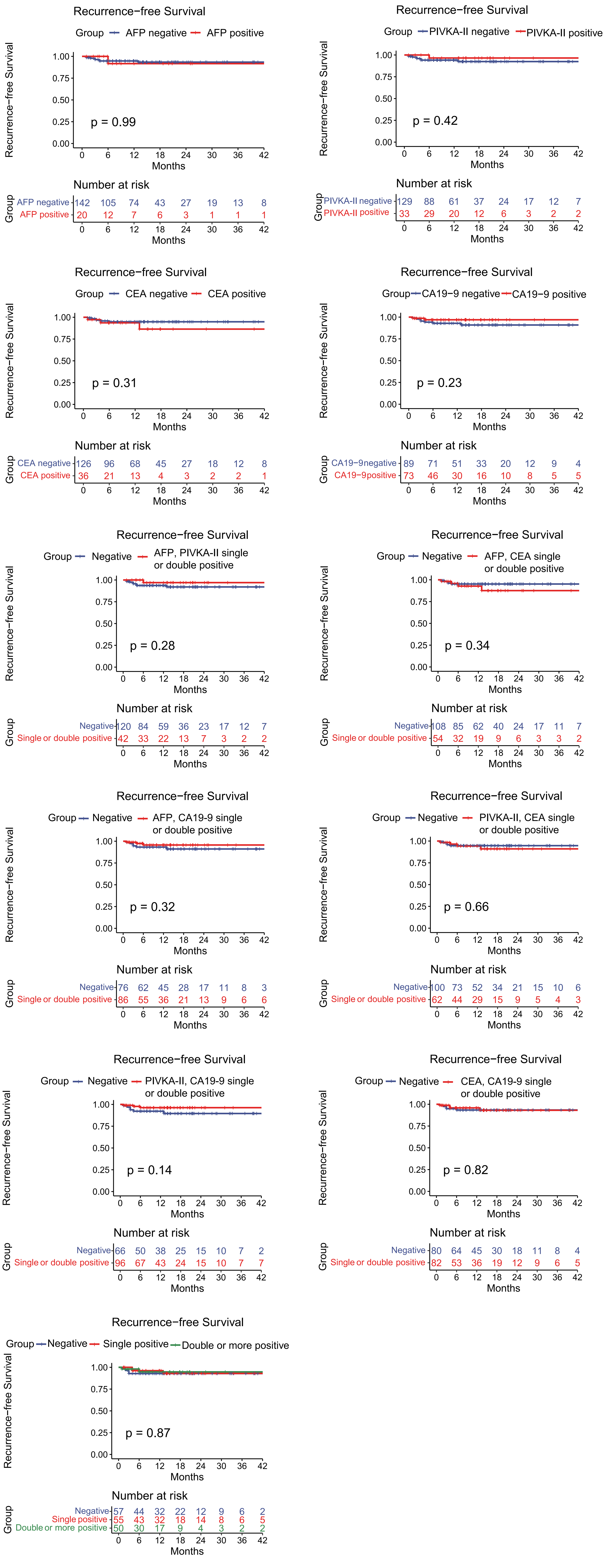

Figure S2. Lymph node metastasis recurrence-free survival curves of grouped intrahepatic cholangiocarcinoma patients based on the (A) AFP, (B) PIVKA-II, (C) CEA, (D) CA19-9, (E) AFP+PIVKA-II, (F) AFP+CEA, (G) AFP+CA19-9, (H) PIVKA-II+CEA, (I) PIVKA-II+CA19-9, (J) CEA+CA19-9, (K) positive numbers of liver tumor marker.
