# Supplementary material for: Liver Tumor Markers, HALP Score, and NLR: Simple, Cost-Effective, Easily Accessible Indexes for Predicting Prognosis in ICC Patients after Surgery
Source: J Pers Med. 2022 Dec 9;12(12):2041. doi: 10.3390/jpm12122041 (PMC9784982; doi:10.3390/jpm12122041)
Supplement: Supplementary file 1 [file jpm-12-02041-s001.zip › Tables S1 and S2.pdf]

Table S1. The association of demographics and clinicopathologic characteristics with different serum tumor markers.

Variables	AFP			CA19-9			CEA			PIVKA-II		
	Positive (N = 20)	Negative (N = 142)	p-Value	Positive (N = 73)	Negative (N = 89)	p-Value	Positive (N = 36)	Negative (N = 126)	p-Value	Positive (N = 33)	Negative (N = 129)	p-Value
Age (y, Mean ± SD)	54.9 ± 12.0	56.9 ± 9.4	0.391	58.0 ± 8.4	55.5 ± 10.6	0.098	58.8 ± 7.7	56.0 ± 10.2	0.125	55.0 ± 10.4	57.0 ± 9.6	0.291
Gender(%male)	19(95.0%)	81 (57.0%)	0.001 *	46(63.0%)	54(60.7%)	0.761	20 (55.6%)	80(63.5%)	0.388	25(75.8%)	75(58.1%)	0.063
Platelet count (x109/L)	232.8 ± 76.7	246.6 ± 84.2	0.486	236.9 ± 81.9	251.5 ± 84.1	0.265	243.7 ± 72.1	245.3 ± 86.4	0.914	249.1 ± 112.9	243.8 ± 74.2	0.746
Albumin (mg/dL)	42.5 ± 3.6	43.7 ± 3.7	0.156	43.0 ± 3.6	44.1 ± 3.6	0.050 *	43.2 ± 3.5	43.7 ± 3.7	0.529	44.4 ± 3.8	43.4 ± 3.6	0.138
Alanine aminotransferase (U/L)	33.9 ± 20.9	35.6 ± 35.1	0.829	37.7 ± 26.4	33.6 ± 38.4	0.443	41.3 ± 27.7	33.8 ± 35.0	0.236	37.3 ± 25.3	34.9 ± 35.5	0.717
Aspartate aminotransferase (U/L)	32.1 ± 12.7	32.7 ± 28.7	0.918	33.6 ± 20.9	31.8 ± 31.6	0.681	36.3 ± 24.6	31.6 ± 28.0	0.360	32.8 ± 24.2	32.6 ± 28.0	0.964
Total bilirubin (umol/L)	14.5 ± 6.2	13.8 ± 21.1	0.890	16.1 ± 29.2	12.1 ± 4.3	0.255	20.0 ± 41.1	12.2 ± 4.8	0.036 *	14.0 ± 8.7	14.4 ± 21.5	0.888
Prothrombin time (s)	12.0 ± 0.9	12.0 ± 5.2	1.000	11.7 ± 1.2	12.3 ± 6.5	0.431	11.8 ± 1.2	12.1 ± 5.5	0.756	11.9 ± 1.0	12.1 ± 4.8	0.788
Hepatis B surface antigen positive (%)	15(75.0%)	70(49.3%)	0.031 *	35(47.9%)	50(56.2%)	0.296	17(47.2%)	68(54.0%)	0.475	22(66.7%)	63 (48.8%)	0.067
Child-Pugh classifications (A/B/C, %)	20(100.0%)/ 0(0%)/ 0(0%)	140(98.6%)/ 2(1.4%)/ 0(0%)	0.593	71(97.3%)/ 2(2.7%)/ 0(0%)	89(100.0%)/ 0(0%)/ 0(0%)	0.116	35(97.2%)/ 1(2.8%)/ 0(0%)	125(99.2%)/ 1(0.8%)/ 0(0%)	0.342	33(100.0%)/ 0(0%)/ 0(0%)	127(98.4%)/ 2(1.6%)/ 0(0%)	0.472
Barcelona Clinic Liver Cancer staging (A/B/C, %)	14(70.0%)/ 4(20.0%)/ 2 (10.0%)	86(60.6%)/ 16(11.3%)/ 40(28.2%)	0.168	40(54.8%)/ 6(8.2%)/ 27(37.0%)	60(67.4%)/ 14(15.7%)/ 15(16.9%)	0.010 *	15(41.7%)/ 5(13.9%)/ 16(44.4%)	85(67.5%)/ 15 (11.9%)/ 26(20.6%)	0.010*	19(57.6%)/ 10(30.3%)/ 4(12.1%)	81(62.8%)/ 10(7.8%)/ 38(29.5%)	0.001 *
AJCC TNM staging system (I/II/III/IV, %)	8 (40.0%)/ 7(35.0%)/ 3(15.0%)/ 2(10.0%)	74(52.1%)/ 34(23.9%)/ 24(16.9%)/ 10(7.0%)	0.659	31(42.5%)/ 16 (21.9%)/ 18(24.7%)/ 8(11.0%)	51(57.3%)/ 25(28.1%)/ 9(10.1%)/ 4 (4.5%)	0.021 *	10(27.8%)/ 12(33.3%)/ 10(27.8%)/ 4(11.1%)	72(57.1%)/ 29(23.0%)/ 17(13.5%)/ 8(6.3%)	0.017 *	13(39.4%)/ 16(48.5%)/ 4(12.1%)/ 0(0%)	69(53.5%)/ 25(19.4%)/ 23(17.8%)/ 12(9.3%)	0.004 *
Tumor differentiations (unknown/well/well-moderate/moderate/moderate-poor/poor, %)	1(5.0%)/ 0(0%)/ 0(0%)/ 5(25.0%)/ 9(45.0%)/	10(7.0%)/ 1(0.7%)/ 1(0.7%)/ 40(28.2%)/ 74(52.1%)/	0.673	7(9.6%)/ 1(1.4%)/ 1(1.4%)/ 20(27.4%)/ 35(47.9%)/	4(4.5%)/ 0(0%)/ 0(0%)/ 25(28.1%/)/ 48(53.9%)/	0.507	4(11.1%)/ 0(0%)/ 0(0%)/ 9(25.0%)/ 19(52.8%)/	7(5.6%)/ 1(0.8%)/ 1(0.8%)/ 36(28.6%)/ 64(50.8%)/	0.833	1(3.0%)/ 0(0%)/ 0(0%)/ 5(15.2%)/ 18(54.5%)/	10(7.8%)/ 1(0.8%)/ 1 (0.8%)/ 40 (31.0%)/ 65(50.4%)/	0.065

	5(25.0%)	16(11.3%)		9(12.3%)	12(13.5%)		4(11.1%)	17(13.5%)		9(27.3%)/	12(9.3%)/	
**Tumor numbers**	12(60.0%)/	96(67.6%)/	0.499	49(67.1%)/	59(66.3%)/	0.911	19(52.8%)/	89(70.6%)/	0.045 *	16 (48.5%)/	92 (71.3%)/	0.013 *
**(single/multiple, %)**	8(40.0%)	46(32.4%)		24(32.9%)	30(33.7%)		17(47.2%)	37(29.4%)		17 (51.5%)	37(28.7%)	
**Main tumor size (cm)**	6.6 ± 3.7	5.7 ± 2.5	0.148	5.7 ± 2.3	6.0 ± 3.0	0.357	6.0 ± 2.8	5.7 ± 2.7	0.662	5.7 ± 3.5	5.8 ± 2.5	0.793
**Main tumor size (≤5.0 cm/>5.0**	8(40.0%)/	67(47.2%)/	0.546	33(45.2%)/	42(47.2%)/	0.801	15(41.7%)/	60(47.6%)/	0.528	19(57.6%)/	56(43.4%)/	0.145
**cm, %)**	12(60.0%)	75(52.8%)		40(54.8%)	47(52.8%)		21(58.3%)	66(52.4%)		14(42.4%)	73(56.6%)	
**Presence vascular invasion (%)**	2(10.0%)	31(21.8%)	0.219	19(26.0%)	14(15.7%)	0.105	12(33.3%)	21(16.7%)	0.029*	7(21.2%)	26(20.2%)	0.893
**Microvascular invasion (%)**	5(26.3%)	38(27.0%)	0.953	19(26.4%)	24(27.3%)	0.900	14(40.0%)	29(23.2%)	0.048 *	10(31.3%)	33(25.8%)	0.533
**Nerve tract invasion (%)**	1(5.3%)	29(20.6%)	0.109	16(22.2%)	14(15.9%)	0.309	11(31.4%)	19(15.2%)	0.030 *	5(15.6%)	25(19.5%)	0.613
**Presence lymph node metastasis (±, %)**	1(5.0%)	26(18.3%)	0.135	17 (23.3%)	10 (11.2%)	0.041 *	9(25.0%)	18(14.3%)	0.128	2(6.1%)	25(19.4%)	0.067
**Presence distant metastasis (±, %)**	1(5.0%)	8(5.6%)	0.908	6(8.2%)	3(3.4%)	0.180	4(11.1%)	5(4.0%)	0.099	0 (0%)	9(7.0%)	0.118
**Overall survival (months)**	19.8 ± 13.2	20.8 ± 12.4	0.744	18.8 ± 13.0	22.2 ± 11.9	0.084	15.6 ± 11.3	22.2 ± 12.4	0.005 *	22.2 ± 10.9	20.3 ± 12.8	0.453
**Recurrence-free survival (months)**	11.9 ± 12.4	15.0 ± 12.4	0.296	12.7 ± 12.4	16.2 ± 12.3	0.078	10.9 ± 11.2	15.7 ± 12.6	0.040 *	15.7 ± 11.2	14.3 ± 12.8	0.578
**Recurrence**	14(70.0%)	80 (56.3%)	0.246	48 (65.8%)	46(51.7%)	0.071	28 (77.8%)	66(52.4%)	0.006 *	18(54.5%)	76(58.9%)	0.650
**Intrahepatic tumor recurrence**	7(35.0%)	52(36.6%)	0.888	28(38.4%)	31(34.8%)	0.643	13(36.1%)	46 (36.5%)	0.965	11(33.3%)	48(37.2%)	0.680
**lymph node metastasis**	1(5.0%)	8(5.6%)	0.908	2(2.7%)	7 (7.9%)	0.156	3(8.3%)	6(4.8%)	0.409	1(3.0%)	8(6.2%)	0.478
**distant metastasis**	2(10.0%)	13(9.2%)	0.903	10(13.7%)	5(5.6%)	0.077	6(16.7%)	9(7.1%)	0.082	3(9.1%)	12 (9.3%)	0.970

Notes: * $p < 0.05$.

Table S2. The association of demographics and clinicopathologic characteristics with different HALP score, PLR, and NLR.

Variables	HALP ≥ 43.63 (N = 100)	HALP < 43.63 (N = 62)	p-Value	PLR ≥ 76.51 (N = 147)	PLR < 76.51 (N = 15)	p-Value	NLR ≥ 3.73 (N = 28)	NLR < 3.73 (N = 134)	p-Value
**Age (y, Mean ± SD)**	56.6 ± 9.5	56.6 ± 10.2	0.993	56.3 ± 10.0	59.8 ± 6.5	0.183	54.8 ± 9.8	57.0 ± 9.7	0.270
**Gender(%male)**	68(68.0%)	32 (51.6%)	0.037 *	88(59.9%)	12(80.0%)	0.126	18(64.3%)	82 (61.2%)	0.760
**Platelet count (x109/L)**	219.5 ± 61.9	285.9 ± 96.3	<0.001 *	252.3 ± 81.2	172.9 ± 68.4	<0.001 *	241.0 ± 98.7	245.7 ± 80.0	0.787
**Albumin (mg/dL)**	44.3 ± 3.2	42.4 ± 4.1	0.002	43.6 ± 3.6	43.2 ± 4.4	0.675	41.7 ± 4.9	44.0 ± 3.2	0.029 *
**Alanine aminotransferase (U/L)**	35.9 ± 26.0	34.6 ± 43.3	0.806	34.0 ± 33.6	49.4 ± 31.7	0.091	32.5 ± 23.7	36.0 ± 35.4	0.618
**Aspartate aminotransferase (U/L)**	30.8 ± 18.2	35.6 ± 37.5	0.282	32.1 ± 27.9	37.9 ± 19.1	0.431	34.9 ± 24.7	32.2 ± 27.8	0.629
**Total bilirubin (umol/L)**	13.1 ± 5.7	15.2 ± 31.5	0.503	14.0 ± 20.8	13.1 ± 4.8	0.874	21.6 ± 46.1	12.3 ± 5.7	0.024 *
**Prothrombin time (s)**	12.3 ± 6.2	11.7 ± 1.1	0.466	12.0 ± 5.1	12.0 ± 1.5	0.964	12.3 ± 1.5	12.0 ± 5.3	0.737
**Hepatis B surface antigen positive (%)**	54(54.0%)	31(50.0%)	0.620	72(49.0%)	13(86.7%)	0.005 *	13(46.4%)	64(47.8%)	0.898
**Child-Pugh classifications (A/B/C, %)**	100(100.0%)/<br>0(0%)/<br>0(0%)	60(96.8%)/<br>2(3.2%)/<br>0(0%)	0.071	145(98.6%)/<br>2(1.4%)/<br>0(0%)	15(100.0%)/<br>0(0%)/<br>0(0%)	0.649	27(96.4%)/<br>1(3.6%)/<br>0(0%)	133(99.3%)/<br>1(0.7%)/<br>0(0%)	0.218
**Barcelona Clinic Liver Cancer staging (A/B/C, %)**	62(62.0%)/<br>16(16.0%)/<br>22(22.0%)	38(61.3%)/<br>4(6.5%)/<br>20 (32.3%)	0.112	88(59.9%)/<br>17(11.6%)/<br>42(28.6%)	12(80.0%)/<br>3(20.0%)/<br>0(0%)	0.051	12(42.9%)/<br>3(10.7%)/<br>13(46.4%)	88(65.7%)/<br>17(12.7%)/<br>29(21.6%)	0.023 *
**AJCC TNM staging system (I/II/III/IV, %)**	52(52.0%)/<br>27(27.0%)/<br>15(15.0%)/<br>6(6.0%)	30(48.4%)/<br>14 (22.6%)/<br>12(19.4%)/<br>6 (9.7%)	0.676	75(51.0%)/<br>34(23.1%)/<br>27(18.4%)/<br>11 (7.5%)	7(46.7%)/<br>7(46.7%)/<br>0(0%)/<br>1 (6.7%)	0.122	9(32.1%)/<br>6(21.4%)/<br>8 (28.6%)/<br>5(17.9%)	73(54.5%)/<br>35(26.1%)/<br>19(14.2%)/<br>7(5.2%)	0.016 *
**Tumor differentiations (unknown/well/well-moderate/moderate/moderate- poor/poor, %)**	6(6.0%)/<br>0(0%)/<br>1(1.0%)/<br>31(31.0%)/<br>51(51.0%)/<br>11(11.0%)/	5(8.1%)/<br>1(1.6%)/<br>0(0%)/<br>14(22.6%)/<br>32(51.6%)/<br>10 (16.1%)/	0.517	11(7.5%)/<br>1(0.7%)/<br>0(0%)/<br>42(28.6%)/<br>75(51.0%)/<br>18(12.2%)/	0(0%)/<br>0(0%)/<br>1(6.7%)/<br>3(20.0%/)/<br>8(53.3%)/<br>3(20.0%)/	0.034 *	3(10.7%)/<br>0(0%)/<br>0(0%)/<br>7(25.0%)/<br>15(53.6%)/<br>3(10.7%)/	8(6.0%)/<br>1(0.7%)/<br>1 (0.7%)/<br>38(28.4%)/<br>68(50.7%)/<br>18(13.4%)/	0.919
**Tumor numbers (single/multiple, %)**	62(62.0%)/<br>38(38.0%)	46(74.2%)/<br>16(25.8%)	0.110	99(67.3%)/<br>48(32.7%)	9(60.0%)/<br>6(40.0%)	0.565	18(64.3%)/<br>10(35.7%)	90(67.2%)/<br>44(32.8%)	0.769

**Main tumor size (cm)**	5.4 ± 2.5	6.4 ± 2.9	0.014 *	6.0 ± 2.7	4.1 ± 1.7	0.011 *	7.0 ± 2.8	5.5 ± 2.6	0.008 *
**Main tumor size (≤ 5.0 cm/ > 5.0 cm, %)**	51(51.0%)/	24(38.7%)/	0.127	65(44.2%)/	10(66.7%)/	0.097	7(25.0%)/	68(50.7%)/	0.013 *
	49(49.0%)	38(61.3%)		82(55.8%)	5(33.3%)		21(75.0%)	66(49.3%)	
**Presence vascular invasion (%)**	19(19.0%)	14(22.6%)	0.582	32(21.8%)	1(6.7%)	0.167	8(28.6%)	25(18.7%)	0.236
**Microvascular invasion (%)**	29(29.0%)	14(23.3%)	0.434	37(25.5%)	6(40.0%)	0.228	5(19.2%)	38(28.4%)	0.337
**Nerve tract invasion (%)**	14(14.0%)	16(26.7%)	0.047*	29 (20.0%)	1 (6.7%)	0.208	5(18.5%)	25(18.8%)	0.973
**Presence lymph node metastasis (±, %)**	14(14.0%)	13(21.0%)	0.247	27 (18.4%)	0(0%)	0.069	9(32.1%)	18(13.4%)	0.016 *
**Presence distant metastasis (±, %)**	4(4.0%)	5(8.1%)	0.272	9(6.1%)	0(0%)	0.324	2(7.1%)	7(5.2%)	0.687
**Overall survival (months)**	20.2 ± 11.6	21.5 ± 13.7	0.497	20.5 ± 12.4	23.0 ± 13.0	0.453	16.5 ± 10.6	21.6 ± 12.6	0.048 *
**Recurrence-free survival (months)**	13.4 ± 10.8	16.6 ± 14.6	0.140	14.3 ± 12.4	18.2 ± 13.0	0.248	7.8 ± 7.0	16.0 ± 12.9	<0.001 *
**Progression**	63(63.0%)	31(50.0%)	0.103	89(60.5%)	5(33.3%)	0.042 *	22 (78.6%)	72(53.7%)	0.015 *
**Intrahepatic tumor recurrence**	43(43.0%)	16 (25.8%)	0.027 *	54(36.7%)	5 (33.3%)	0.794	12(42.9%)	47(35.1%)	0.436
**lymph node metastasis**	4(4.0%)	5(8.1%)	0.272	8(5.4%)	1 (6.7%)	0.844	5(17.9%)	4(3.0%)	0.002 *
**distant metastasis**	8(8.0%)	7(11.3%)	0.483	14(9.5%)	1 (6.7%)	0.716	4 (14.3%)	11(8.2%)	0.313
